# Simultaneous renal clear cell carcinoma and primary clear cell carcinoma of the liver: A case report

**DOI:** 10.1097/MD.0000000000023263

**Published:** 2020-11-20

**Authors:** Hua Jiang, Shanchao Zhao, Ganhong Li

**Affiliations:** a Department of Urology, The Fifth Affiliated Hospital of Zunyi Medical University, Zhuhai; b Department of Urology, Nanfang Hospital, Southern Medical University/The First School of Clinical Medicine, Southern Medical University, Guangzhou; c Department of Urology, The Fifth Affiliated Hospital of Zunyi Medical University, Zhuhai, China.

**Keywords:** adrenal gland, metastasis, primary clear cell carcinoma of the liver, renal clear cell carcinoma

## Abstract

**Rationale:**

Double primary clear cell carcinomas of the liver (PCCCL) and kidney are extremely rare; moreover, there have been no reported cases of adrenal metastasis from primary clear cell tumors of the liver.

**Patient concerns:**

A 47-year-old male patient was admitted to our clinic with space-occupying lesions in the left kidney and liver during a regular medical examination.

**Diagnoses:**

The tumors in the kidney and liver were diagnosed as primary clear cell carcinoma by histopathological examination.

**Interventions:**

The patient subsequently underwent nephron-sparing surgery of the left kidney and radical partial excision of the right liver lobe by laparoscopic surgery. Transcatheter arterial chemoembolization (TACE) was performed for the patient 2 weeks after tumor resection. One month after the operation, the patient started adjuvant therapy with sorafenib (400 mg twice per day orally). However, follow-up CT imaging revealed a solid mass measuring 1.9 × 2.0 × 2.0 cm^3^ in the right adrenal gland at 2 months postoperatively, and then the patient underwent radiofrequency ablation (RFA) for the right adrenal tumor.

**Outcomes:**

The patient remained cancer free for 2 years following the diagnosis despite early right adrenal metastasis.

**Lessons:**

Hepatocyte immunostaining is sufficient for the diagnosis of PCCCL.

## 1 Introduction

Primary clear cell carcinoma is a common type of renal and ovarian cancer, especially renal carcinoma, and renal clear cell carcinoma (RCCC) accounts for 85% of all kidney tumors.^[1]^ PCCCL is a rare tumor with few cases reported worldwide thus far, and simultaneous clear cell carcinoma of the kidney and liver is exceedingly rare. It is generally believed that PCCCL has a better survival rate than nonclear cell hepatocellular carcinoma due to its low incidence and unclear clinicopathological characteristics. There are no reports of PCCCL metastasis to the adrenal gland in the literature. We herein report a case of simultaneous double RCCC and PCCCL in a 47-year-old man with right adrenal metastasis from postoperative PCCCL.

## 2 Case report

### 2.1 Clinical course

**Figure 1:**
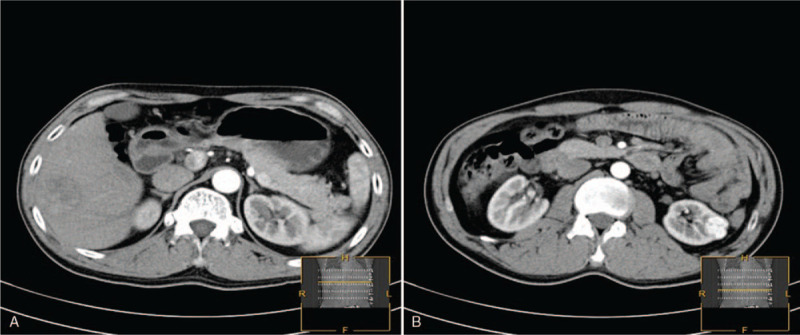
CT prior to nephron-sparing surgery of the left kidney and radical partial excision of the right liver lobe. (A) The enhanced computed tomography (CT) scan revealed a mildly enhanced tumor measuring approximately 3 × 4 × 5 cm^3^ in the right lobe of the liver. (B) Another significantly enhanced mass measuring approximately 2 × 3 × 3 cm^3^ was observed in the inferior pole of the left kidney without local invasion or lymph node metastases, and no mass lesions were found on the bilateral adrenal glands.

A 47-year-old man was admitted to the Department of Urology of the Fifth Affiliated Hospital of Zunyi Medical College with space-occupying lesions in the kidney and liver during a regular medical examination in August 2016. The patient complained of mild, intermittent, right upper quadrant discomfort for 2 months. Moreover, the patient had a history of chronic hepatitis B for 20 years. Physical examination showed that the liver edge was palpable 3 cm below the right costal margin and that there was mild tenderness. There was no yellowing of the skin or mucosa and no liver palms. The complete blood count and serum biochemistry data on admission were as follows: hemoglobin, 136 g/l (normal range, 120∼160 g/l); white blood cell, 4.2 × 10^9^/l (normal range, 4.0–10 × 10^9^/l); platelets, 154 × 10^9^/l (normal range, 100∼300 × 10^9^/l); alpha-fetoprotein (AFP), 35.49 ng/ml (normal range, 0∼7 ng/m); glucose (fasting) 5.0 mmol/l (normal range, 3.9∼6.1 mmol/l); normal protein/albumin 42.6 g/l (normal range, 40∼55 g/l) and 25.0 g/l (normal range, 20∼40 g/l); a normal bilirubin 8.0 μmol/l (normal range, 0∼23 μmol/l). His liver enzymes were within the normal range (ALT 21 units/l, AST 16 units/l). The hepatitis virus test showed positivity for hepatitis B surface antigen (HBsAg), but he was negative for HCV. The enhanced computed tomography (CT) scan revealed a mildly enhanced tumor measuring approximately 3 × 4 × 5 cm^3^ in the right lobe of the liver and an obviously enhanced mass measuring approximately 2 × 3 × 3 cm^3^ in the inferior pole of the left kidney, and no mass lesions were found on the bilateral adrenal glands (Fig. 1). Enlargement of the lymph nodes was not found in the abdominal cavity or retroperitoneum.

### 2.2 Therapeutic intervention and immunohistochemical (IHC) analyses

**Figure 2:**
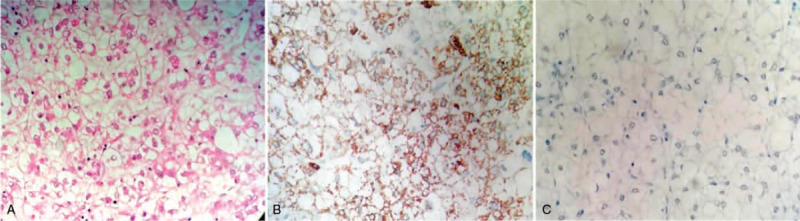
HE and immunological staining of the hepatic tumor: (A) Bulky cancer cells with entirely or almost entirely “clear” cytoplasm were found in the tumor (original magnification × 400); (B) hepatocyte staining revealed strong positivity in the tumor cells (original magnification × 400); (C) CD10 staining of the tumor cells was completely negative (original magnification × 400).

**Figure 3:**
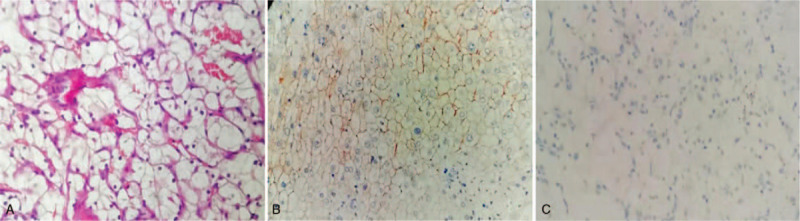
HE and immunological staining observation of the renal tumor: (A) Sheet of clear cells in the tumor (original magnification × 400); (B) CD10 staining revealed diffuse positivity among the tumor cells (original magnification × 400); (C) hepatocyte staining of tumor cells was completely negative (original magnification × 400).

The clinical presumptive diagnoses were RCCC (T1N0M0) and HCC (T2N0M0). The patient subsequently underwent nephron-sparing surgery (NSS) of the left kidney and radical partial excision of segments Vand VI of the right liver lobe by laparoscopic surgery. The IHC findings of the PCCCL (Fig. 2) were as follows: hepatocyte (+), AFP (+), CEA (-), and HBsAg (+). The pathological findings of the renal tumor revealed Fuhrman grade II RCCC (Fig. 3), and the PCCCL was stage B according to the Barcelona Clinic Liver Cancer (BCLC) staging system. The immunohistochemistry results of the kidney tumor were as follows: CD10 (+), CK7 (-), EMA (+), and vimentin (+). Based on the radiological imaging and pathological results, neither regional lymph node nor distant lymph node metastasis was found, and the TNM (tumor-node-metastasis) classification was T1N0M0 for RCCC and T2aN0M0 for PCCCL. TACE was performed in the segmental artery 2 weeks after tumor resection. One month after the operation, the patient started adjuvant therapy with sorafenib (400 mg twice per day orally), and the re-examination of serum AFP showed a level of 5.22 ng/m.

**Figure 4:**
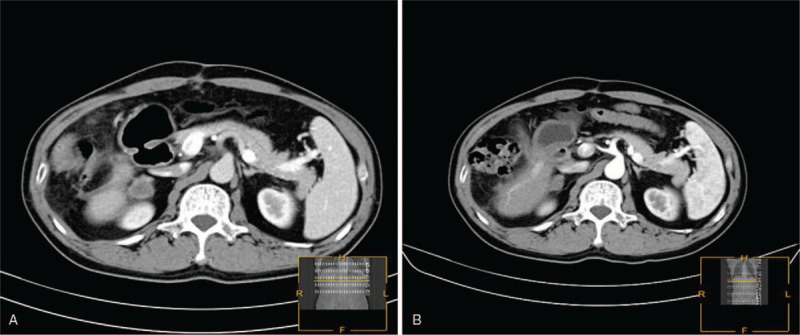
Images of the right adrenal metastasis 2 months after radical partial excision of the right liver lobe. (A) Enhanced CT scan shows a tumor in the right adrenal gland; (B) 3 months after radiofrequency ablation, the tumor had been completely ablated and devascularized, with the disappearance of tumor enhancement.

Unfortunately, a follow-up CT image revealed a 1.9 × 2.0 × 2.0 cm^3^ mass on the right adrenal gland 2 months postoperatively (Fig. 4A). His serum AFP was 13.27 ng/ml, and the patient underwent radiofrequency ablation (RFA) for right adrenal metastasis. The patients postoperative course was uneventful, and he was discharged after 14 days. He was followed up regularly with clinical examinations and tumor marker tests (including those for AFP, CEA, CA-199, and CA125) every 3 months. No local recurrence or distant metastasis was found on the follow-up abdominal CT scans, which were acquired every 3 months for 2 years.

## 3 Discussion

This is a case of simultaneous renal clear cell carcinoma and primary clear carcinoma of the liver. These conditions are consistent with the definition of multiple primary malignant neoplasms (MPMNs). MPMNs were first described by Billroth in 1889, and the diagnostic criteria were established by Warren and Gates in 1932. MPMNs are defined as synchronous or metachronous tumors in 1 individual. The occurrence of multiple primary cancers in a single patient is relatively rare. The prevalence of MPMNs has been reported to vary from 0.734% to 16% in various research and across different countries.^[2–4]^ The molecular mechanism of MPMN, however, remains unclear, and some putative risk factors have been implicated in its pathogenesis, including older age,^[5,6]^ exposure to chemotherapy and radiotherapy,^[3,4,7]^ genetic mutation,^[8,9]^ and a history of tobacco and alcohol use,^[10,11]^ and several shared pathways^[12]^ are likely to contribute to the development of MPMNs.

There are no sufficient epidemiological data about primary clear cell carcinoma of the liver due to the rarity of this kind of cancer, and it is generally considered that PCCCL has a better survival rate than nonclear cell hepatocellular carcinoma.^[13]^ PCCCL is a specific and uncommon variant of HCC, with an incidence of 0.4% to 12.5% among all liver cancers reported in the published literature.^[14–16]^ Primary clear cell carcinomas arise in many organs (kidney, ovaries, thyroid or liver) and can mimic other types of tumors. It is difficult to distinguish PCCCL from other common clear cell carcinomas merely by morphological characteristics.^[17,18]^ It has been reported that positive hepatocyte immunostaining is sufficient for a diagnosis of PCCCL; hepatocyte immunohistochemistry could distinguish PCCCL from other clear cell cancers with a specificity of 100% and sensitivity of 90%,^[17]^ and immunohistochemical staining revealed strong positive staining for cluster of differentiation marker 10 (CD10) and vimentin, which is consistent with the diagnosis of RCCC.^[19]^

The adrenal gland is an uncommon site for metastasis from primary liver tumors, and the adrenal glands are reportedly the fourth most frequent site of extrahepatic metastasis from HCC.^[20,21]^ To the best of our knowledge, it is extremely rare for both the kidney and liver to develop clear cell carcinomas, and only 2 cases have been reported in the literature.^[22,23]^ However, there is no report of adrenal metastasis from PCCCL that coexists with RCCC in the literature.

In our case, immunohistochemical staining showed that the hepatic tumor cells were diffusely positive for hepatocytes and negative for CD10. Clear cell renal carcinoma was positive for CD10 but negative for hepatocytes. Based on the literature report and immunohistochemical results, a final diagnosis of simultaneous clear cell carcinoma of the left kidney and liver was confirmed.

Although the adrenal gland is the fourth most common site of metastasis from HCC, the underlying mechanism of adrenal metastasis from HCC is unclear. According to Okuda,^[24]^ the right adrenal gland is occasionally found to be tightly adherent to the inferior surface of the right liver, forming what is called adrenohepatic fusion. According to autopsy results,^[25,26]^ 10% of individuals had anatomical variations, and the incidence increased rapidly above the age of 60 years; thus, tumor cells could metastasize to the right adrenal gland through ectatic blood vessels of the adrenohepatic fusion.

Although patients with adrenal metastasis from HCC have a poor prognosis, it has been reported that adrenal metastasis should be treated actively, and patients who receive adrenalectomy, TACE, percutaneous ethanol injection (PEI), radiotherapy, or radiofrequency ablation (RFA) treatment always have better survival than those without treatment.^[27–31]^ Kim et al. showed that the overall actuarial 5-year survival rate was 24%, with a median survival of 21 months after adrenalectomy.^[32]^ Furthermore, recent reports have described successful radiofrequency ablation for adrenal metastasis from lung cancer, renal cell carcinoma, colorectal cancer, hepatocellular carcinoma, and other tumors; the overall 5-year survival rate was 30%, with a median survival time of 26 months.^[29,33]^ This patient received RFA for adrenal metastasis, and the tumor was completely ablated and devascularized, with the disappearance of tumor enhancement 3 months after RFA (Fig. 4B). No local recurrence or distant metastasis have been detected on the follow-up abdominal CT scans as of yet.

In this study, we reported a case of simultaneous RCC and PCCCL with adrenal metastatic disease as well as comprehensive treatment including NSS, radical partial excision of the right liver, TACE for PCCCL, systemic chemotherapy, and RFA for the metastatic mass in the right adrenal gland. Double primary clear cell carcinomas of the liver and kidney are extremely rare, and it is difficult to distinguish PCCCL from RCC. Hepatocyte immunostaining is sufficient for the diagnosis of PCCCL, and hepatocyte immunohistochemistry could distinguish PCCCL from other clear cell cancers with good specificity and sensitivity. Although adrenal metastasis has a poor outcome worldwide, active treatment of adrenal metastasis with surgical or nonsurgical methods may provide a favorable survival rate.

## Author contributions

**Conceptualization:** Hua Jiang, Shanchao Zhao, Ganhong Li.

**Formal analysis:** Hua Jiang.

**Investigation:** Hua Jiang, Shanchao Zhao, Ganhong Li.

**Resources:** Hua Jiang.

**Writing – original draft:** Hua Jiang.

**Writing – review & edits:** Hua Jiang, Shanchao Zhao.
